# Mother-to-Infant Microbial Transmission from Different Body Sites Shapes the Developing Infant Gut Microbiome

**DOI:** 10.1016/j.chom.2018.06.005

**Published:** 2018-07-11

**Authors:** Pamela Ferretti, Edoardo Pasolli, Adrian Tett, Francesco Asnicar, Valentina Gorfer, Sabina Fedi, Federica Armanini, Duy Tin Truong, Serena Manara, Moreno Zolfo, Francesco Beghini, Roberto Bertorelli, Veronica De Sanctis, Ilaria Bariletti, Rosarita Canto, Rosanna Clementi, Marina Cologna, Tiziana Crifò, Giuseppina Cusumano, Stefania Gottardi, Claudia Innamorati, Caterina Masè, Daniela Postai, Daniela Savoi, Sabrina Duranti, Gabriele Andrea Lugli, Leonardo Mancabelli, Francesca Turroni, Chiara Ferrario, Christian Milani, Marta Mangifesta, Rosaria Anzalone, Alice Viappiani, Moran Yassour, Hera Vlamakis, Ramnik Xavier, Carmen Maria Collado, Omry Koren, Saverio Tateo, Massimo Soffiati, Anna Pedrotti, Marco Ventura, Curtis Huttenhower, Peer Bork, Nicola Segata

**Affiliations:** 1 Centre for Integrative Biology, University of Trento, 38123 Trento, Italy; 2 European Molecular Biology Laboratory, Structural and Computational Biology Unit, 69117 Heidelberg, Germany; 3 Azienda Provinciale per i Servizi Sanitari, 38123 Trento, Italy; 4 NGS Facility, Laboratory of Biomolecular Sequence and Structure Analysis for Health, Centre for Integrative Biology, University of Trento, 38123 Trento, Italy; 5 Laboratory of Probiogenomics, Department of Chemistry, Life Sciences and Environmental Sustainability, University of Parma, 43124 Parma, Italy; 6 GenProbio srl, 43124 Parma, Italy; 7 Broad Institute of MIT and Harvard, Cambridge, MA 02142, USA; 8 Institute of Agrochemistry and Food Technology, National Research Council, Paterna, 46980 Valencia, Spain; 9 Faculty of Medicine, Bar Ilan University, Safed 1311502, Israel; 10 Department of Biostatistics, Harvard T.H. Chan School of Public Health, Boston, MA 02115, USA

**Keywords:** infant microbiome, shotgun metagenomics, strain-level profiling, microbiome transmission

## Abstract

The acquisition and development of the infant microbiome are key to establishing a healthy host-microbiome symbiosis. The maternal microbial reservoir is thought to play a crucial role in this process. However, the source and transmission routes of the infant pioneering microbes are poorly understood. To address this, we longitudinally sampled the microbiome of 25 mother-infant pairs across multiple body sites from birth up to 4 months postpartum. Strain-level metagenomic profiling showed a rapid influx of microbes at birth followed by strong selection during the first few days of life. Maternal skin and vaginal strains colonize only transiently, and the infant continues to acquire microbes from distinct maternal sources after birth. Maternal gut strains proved more persistent in the infant gut and ecologically better adapted than those acquired from other sources. Together, these data describe the mother-to-infant microbiome transmission routes that are integral in the development of the infant microbiome.

## Highlights

• Strain-resolved metagenomics was used to track mother-to-infant microbiome transfer
• Microbial strains from multiple maternal body sites transfer to the infant microbiome
• The early microbial diversity in the infant gut is rapidly shaped by niche selection
• The maternal gut microbiome is the source of the majority of transmitted strains

## Graphical Abstract

**Figure:**
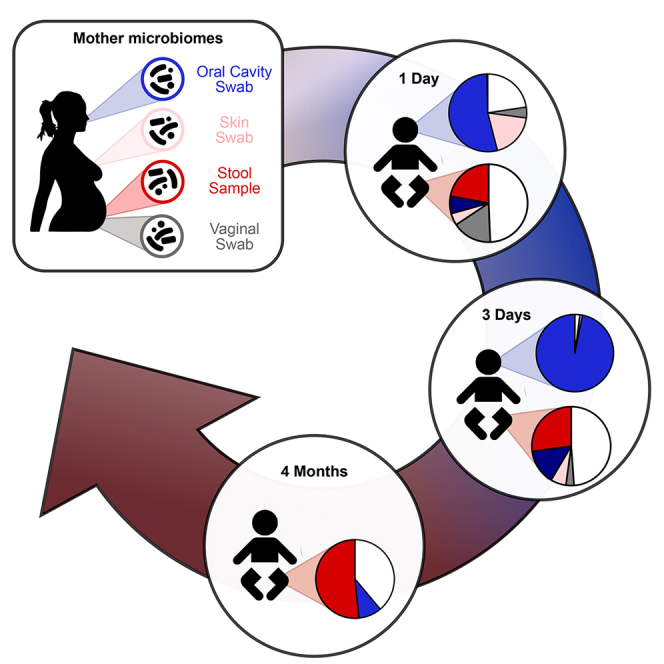

## Teaser

Ferretti et al. use metagenomics with strain-resolved computational profiling to characterize the transfer of microbes from mothers to their infants during their first 4 months of life. Multiple maternal body sites contribute to the developing infant microbiome, with maternal gut strains providing the largest contribution of colonizing microorganisms.

## Introduction

The complex microbial community that inhabits humans, the microbiome, is an integral aspect of human health. In what is undoubtedly a complex interplay between host genetics and environmental conditions, these resident microbes support many functions in the human body, including the facilitation of nutrient absorption that would otherwise be inaccessible to the host (Flint et al., 2012, Wasielewski et al., 2016), the training and modulation of the immune system (Kau et al., 2011, Thaiss et al., 2016), and protection against pathogenic organisms (Buffie and Pamer, 2013). Dysbiosis of this harmonious relationship has reported to be linked to many diseases in adults, including inflammatory bowel diseases (IBDs) (Morgan et al., 2012, Parekh et al., 2015), type 2 diabetes (Qin et al., 2012, Upadhyaya and Banerjee, 2015), and colorectal cancer (Kostic et al., 2012, Vogtmann et al., 2016, Zeller et al., 2014). Similarly, in infants it is associated with IBD (Kolho et al., 2015), Crohn's disease (Gevers et al., 2014, Wang et al., 2016), type 1 diabetes (Kostic et al., 2015), necrotizing enterocolitis (Elgin et al., 2016, Ward et al., 2016), and asthma (Attar, 2015). While the importance of the host-microbiome interplay is not in question, the mechanisms by which an infant acquires these microbes, and from what source, remain largely unexplored.

The long-held belief that an infant is sterile at birth (Escherich, 1988) has been challenged by an increasing number of reports offering evidence of the occurrence of intra-uterine seeding (Aagaard et al., 2014, Perez-Munoz et al., 2017, Rodriguez et al., 2015), but the role and importance of prenatal microbial colonization are still open to debate (Jimenez et al., 2008, Perez-Munoz et al., 2017, Walker et al., 2017). What is clearer is that extensive microbial colonization begins postpartum. Several crucial factors have been linked to the early development of the infant microbiome, including the mode of delivery (Dominguez-Bello et al., 2010, Dominguez-Bello et al., 2016) and gestational age at birth (La Rosa et al., 2014), as well as other influencing factors including maternal and infant antibiotic usage (Lemas et al., 2016, Yassour et al., 2016) and feeding method (formula or breastfeeding) (Backhed et al., 2015). Wider environmental exposure (Shin et al., 2015) and early intimate relations, particularly with the mother (Asnicar et al., 2017, Backhed et al., 2015, Korpela et al., 2018, Nayfach et al., 2016), also play a pivotal role in the early microbial acquisition and community succession in the infant.

Much of what is known about the acquisition of the early infant microbiome has been obtained using cultivation-based (Makino et al., 2011) or taxonomic profiling limited to a species-level resolution (Backhed et al., 2015). As individuals frequently share common species, inferring transmission at lower taxonomic resolutions, even at the species level, is not sufficient as a species can comprise multiple subspecies strain variants, which can be specific to different individuals (Backhed et al., 2015, Truong et al., 2017). It is essential, therefore, to use strain-level profiling to identify and quantify the instances of transmission from external sources to the infant. This has been shown for a limited number of cultivable species (Makino et al., 2011, Milani et al., 2015), and we previously demonstrated that the maternal microbial reservoir is an important source in the early acquisition of microbial species and strains in the infant gut (Asnicar et al., 2017, Korpela et al., 2018). Yet there has been no comprehensive assessment of the multiple potential maternal sources of microbial transmission, and how ultimately they contribute to the acquisition of the infant microbiome within hours of birth and over the first few months of life.

To this end, we investigated what is arguably the most important intimate relationship in the development of the early infant microbiome, the mother (Mueller et al., 2015). Focusing on five potential maternal sources of microbial transmission (skin, breast milk, fecal, vaginal, and oral), 25 mother and infant pairs were recruited and the infants longitudinally sampled from birth up to the first 4 months of life. Using high-resolution shotgun metagenomics (Quince et al., 2017) with improved strain-level computational profiling of known and poorly characterized microbiome members (Segata, 2018), we followed the transmission and assessed the impact of the maternal microbiomes on the development of infant oral and fecal microbial communities from birth to 4 months of life.

## Results

### A Metagenomic Framework to Study Vertical Microbial Transmission

**Figure 1:**
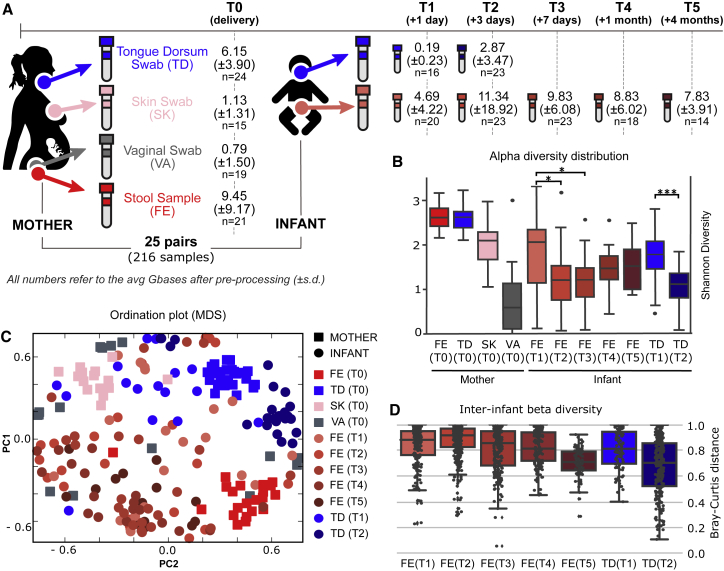
Longitudinal Metagenomic Sequencing of the Microbiome of Mother-Infant Pairs (A) Samples were collected from 25 mother-infant pairs and metagenomically sequenced. Samples were taken from the stool (FE), skin (SK), vagina (VA), and tongue dorsum (TD) of the mothers and from the stool and tongue dorsum of the infants. Sampling of the infant started within 24 hr from delivery and continued for up to 4 months (STAR Methods). All samples were shotgun sequenced and the average depth (in Gbases) of the quality-controlled and human DNA-free samples are reported. (B) Alpha-diversity distributions for each sample type and time point (^∗^p < 0.05, ^∗∗∗^p < 0.001). (C) Ordination plot (MDS) of all the samples that passed preprocessing based on the Bray-Curtis distance between samples highlights the spatial clustering of samples with respect to both different body sites and longitudinal time points. (D) Beta diversities (Bray-Curtis on log-scaled relative abundances) between samples within each infant body site (gut and tongue dorsum) across time points.

We enrolled 25 healthy pregnant women who vaginally delivered healthy newborns at full term. For each mother, we sampled the stool as a proxy of the lower intestine, and four additional body sites: the oral cavity (tongue dorsum swabs), the skin (intermammary cleft swabs), the vagina (vaginal introitus swabs), and the breast milk. Each newborn was sampled at two sites, the gut and the oral cavity, from birth up to 4 months postpartum (Figure 1A). All infants were exclusively breastfed at 3 days, 96% at 1 month, and 56% at 4 months. Of the 44% of non-exclusively breastfed infants at 4 months, 16% of the infants were exclusively formula-fed (Table S1A). All samples were shotgun sequenced, yielding a total of 216 high-quality metagenomes (STAR Methods), with an average of 5.73 (±7.26) Gbases per sample after quality control. DNA extraction from breast milk was not feasible in the majority of the cases, and when enough DNA was recovered a large fraction was found to be of human origin; thus, no high-quality milk microbiome samples were retained for further analysis.

Quantitative taxonomic profiling performed with MetaPhlAn2 (Truong et al., 2015) revealed that the diversity (Figure 1B), structure (Figure 1C), and composition (Figure S1) of the microbial communities are, as previously described (Human Microbiome Project Consortium, 2012a), distinct at each body site. The diversity of the infant microbiome was significantly lower than all the maternal microbiomes except for the *Lactobacillus*-dominated vaginal microbiome (Figure 1B), which is known to have a low diversity (Gajer et al., 2012, Human Microbiome Project Consortium, 2012b, Ravel et al., 2011), and was confirmed by rarefaction analysis to account for variance in sequence depths (Figure S2A). The gut and tongue microbiomes in infants were instead found to have a very high inter-subject variability, particularly at early time points (Figure 1D), compared with that of the mothers (Figure S2B). Interestingly, although the infants were all vaginally delivered, clustering based on initial sampling revealed that the early infant microbiomes did not consistently resemble one specific maternal body site (Figure 1C). For example, in some cases the early infant fecal samples (at 1 and 3 days) clustered with the maternal vaginal samples, while in other instances they clustered with the mothers' fecal samples. The high inter-subject diversity and lack of uniformity in the composition of the infant microbiomes at 1 day of life suggest that the initial exposure and seeding of the microbiome is largely stochastic, with each infant being influenced to a varying degree by the different maternal microbiomes (i.e., vagina, skin, oral, fecal), by potential prebirth *in utero* microbial acquisition, or by contact with other environmental sources.

### Early Acquisition of Microbial Diversity in the Infant Is Subject to Subsequent Rapid Niche Selection

We observed a high species diversity in the infant fecal microbiomes at the first time point (T1, within 24 hr of birth; Figure 1B). This diversity decreased over the first week postpartum (p < 0.05 for both T2 and T3) before recovering over time (Figure 1B). Interestingly, the gut microbiome of exclusively breastfed infants at T4 (4 months) showed a lower diversity than the ones that gradually switched to formula feeding (Figure S2C). The high diversity at day 1 (T1) reflects the rapid influx of microbes, the pioneering microbiome, from maternal as well as other environmental sources after birth, and is consistent with a previous report using 16S rRNA taxonomic profiling (Wampach et al., 2017). Some of these microbes are present only transiently in some infants (Table S2A) as they are probably poorly adapted or unsuited to colonize the infant lower gastrointestinal tract and, as such, are easily lost or replaced. Others are instead more likely to be true early colonizers. In particular, we observed that some species (e.g., *Alistipes putredinis*, *Clostridium innocuum*, *Haemophilus parainfluenzae*, *Prevotella melaninogenica*, and *Streptococcus parasanguinis*) found in the infant stool at day 1 (T1) were not present at subsequent time points (see Table S2A for the full list). This was expected as *P. melaninogenica*, *C. innocuum*, and *Lactobacillus crispatus* are not typically associated with the infant fecal microbiome, never being found to constitute more than 1% abundance in any of the three metagenomic datasets (Backhed et al., 2015, Kostic et al., 2015, Vatanen et al., 2016) available for infants in *curatedMetagenomicData* (Pasolli et al., 2017) (Table S2B). Among the species that were lost in the infants between day 1 and the subsequent time points, 80% were shared with at least one body site of the respective mother (Table S2A). Many of these shared but transient microbes most likely originated from maternal body sites other than the maternal stool (11 times from tongue dorsum, 1 from vagina, and 5 from skin), which could suggest their unsuitability to colonize the infant gut. In contrast, other more typical fecal species (e.g., *Bacteroides vulgatus*, *Bifidobacterium longum*, and *Bifidobacterium breve*) persisted from birth up to at least 4 months of age, when sample collection ended, suggesting that they are indeed colonizing the infant gut (Duranti et al., 2017, Milani et al., 2015).

Prepartum, the infant is subjected to largely anaerobic conditions *in utero*, but at birth the infant gut is mostly facultative aerobic, i.e., oxygen permissive (Houghteling and Walker, 2015, Rodriguez et al., 2015). At day 1 (T1), we found more strict anaerobic species (on average 4.7 species per sample) than aerobic (0.4) or facultative aerobic species (4.4), albeit present at rather low abundances (16.2% in relative abundance; Figure S3). Of these strict anaerobes, 40% of the species were in common with the corresponding mother. On the third day (T2), these strict anaerobes had drastically decreased in number by 26% in the stool and 75% in the tongue dorsum. Over the same period, the abundance of facultative anaerobes increased in the stool (+9.1%) and slightly decreased in the tongue dorsum (−6.3%). These facultative anaerobes are known early colonizers of the infant gut, and these pioneering species mediate the shift from aerobic to anaerobic conditions typically associated with the adult state (Backhed et al., 2015, Houghteling and Walker, 2015, Rodriguez et al., 2015). Probably as a result of this transition toward anaerobic conditions, starting from the first week of age (T3), the number and the relative abundance of strictly anaerobic species increased over time (Figure S3).

This process of early and rapid acquisition of microbial species followed by selection and succession is reflected by inter-infant microbiome distances, which, after the third day, show a decrease as the infant microbiomes converge toward a more defined composition (Figure 1D). This is explained by the decreasing effect of the original direct seeding of the infant microbiome from different maternal body sites in different mother-infant pairs. Nevertheless, the infant gut microbiome at 4 months is still markedly different from that of the mother (Permanova on Bray-Curtis dissimilarity, p < 0.0001), confirming that a longer time window is required to fully appreciate the maturation process from infant to an adult-like state (Koren et al., 2012, Yatsunenko et al., 2012).

### Infants Are Enriched with Microbial Species Present in the Microbiomes of Their Mothers

**Figure 2:**
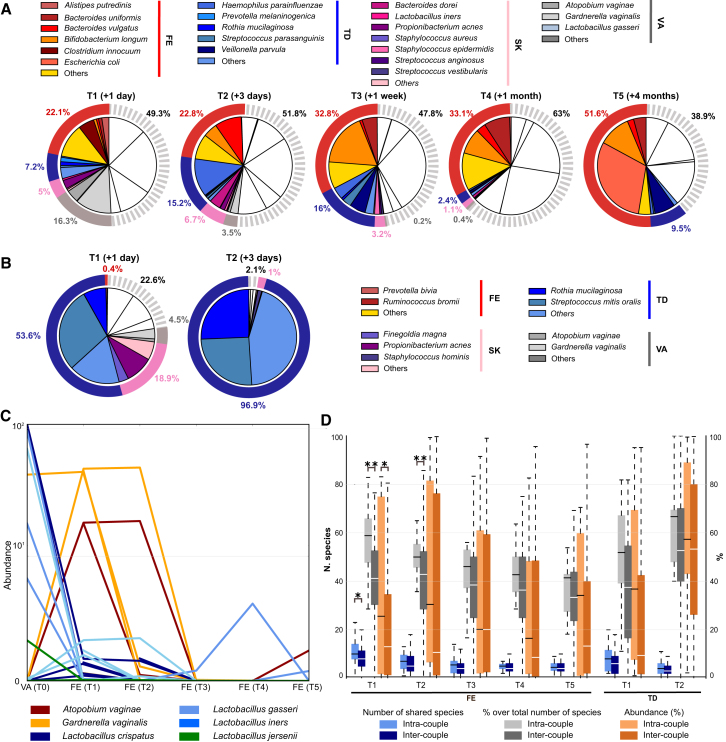
Microbial Species Common to the Mothers and Their Infants (A and B) Average taxonomic composition of the infant stool (A) and tongue dorsum (B) microbiomes over time. The colored sectors indicate species that are found in the infant and his/her mother. White portions refer to species not found in the maternal body sites. External rings show the cumulative abundance of bacterial species per maternal body site. (C) Relative abundances of the most abundant vaginal bacteria in the mothers and in the gut of their infants. Each line represents a mother-infant pair. (D) Number, percentage over total number, and cumulative abundance of identified microbial species that are shared between each mother and her own infant (intra-pair), and between each mother and unrelated infants (inter-pair) (^∗^p < 0.05 and ^∗∗^p < 0.01, t test).

The infant gut microbiomes displayed a large proportion of species in common with their mothers for all mother-infant pairs. At day 1 (T1), about 50% of the microbial population in the infant gut belonged to species also present in at least one of the sampled maternal body sites (Figure 2A), and this fraction was relatively stable over time (50.7% at day 1 [T1], 48.3% at day 3 [T2], 52.2% at 1 week [T3], 37% at 1 month [T4], and 61.2% at 4 months [T5]). Shared species were notably in lower relative abundance in the mothers compared with their infants (between 2.7% and 5.5% in the mothers, depending on the reference infant time point). This suggests that it is the fitness of the microbial organisms reaching the infant gut that plays a greater role than the quantitative contribution of microbial seeding occurring for each species. The species observed in the infants but not shared with their respective mothers are likely to have been acquired from environmental sources, including other individuals that have had contact with the infant (Korpela et al., 2018). In the oral cavity, the relative abundance of shared species between infants and any maternal microbiomes was even more pronounced with 77.6% (day 1, T1) and 95.4% (day 3, T2) of the infant microbiome being shared with the mother (Figure 2B). Also in this case, the shared species were present at low relative abundances in the maternal tongue dorsum compared with the infants (5.7% at day 1 [T1] and 6.6% at day 3 [T2]).

We then considered the species shared between the infant and their mothers, but not with the other mothers in the cohort. Infants at day 1 (T1) shared significantly more microbial species with their mothercompared with other mothers (p < 0.05; Figure 2D). This was confirmed when looking at the fraction of shared species over the total number of species (p < 0.01) and at the cumulative relative abundance of the shared species (p < 0.05; Figure 2D). At 3 days of age (T2), infants still significantly (p < 0.01) shared a larger fraction of species with their mother compared with others, before gradually losing their species-level remembrance of the maternal microbiome (Figure 2D).

To identify the source of the infant microbiome, we then analyzed separately the four sequenced maternal microbiomes as potential reservoirs of microbial transmission. A reanalysis of the Human Microbiome Project data (Human Microbiome Project Consortium, 2012b) confirmed that the compositions of the adult microbiomes belonging to the four body sites considered here are very distinct, with no species shared at comparable abundances across body sites, and this allowed us to compute the lists of body site-specific microbes reported in Table S3. All the maternal body sites contributed to the common mother-infant species with the mothers' stool microbiome accounting for 22.1% of the overall microbial abundance in the infant gut followed by the vagina (16.3%), the oral cavity (7.2%), and the skin (5%). Over time, the abundance of typical vaginal, oral, and cutaneous species decreased, suggesting that these species are likely transient inhabitants of the lower gastrointestinal tract. For instance, most of the vaginal species, which constituted up to 16.3% of the total abundance in the infant stool at day 1 (T1), were either lost or at undetectable levels by 1 week of age (T3). Interestingly, while abundant in the vaginal community, lactobacilli were rarely identified in the infant guts (Figure 2C). This is perhaps a consequence of lactobacilli being acidophilic, preferring a pH of approximately 3.5 (Ma et al., 2012), and the infant gut being near neutral (Evans et al., 1988). This might also explain the persistence of the more pH-tolerant (pH 5.5) vaginal species, *Gardnerella vaginalis* and *Atopobium vaginae*, at least over the first days of life (Figure 2C), although other explanations involving for example nutrient requirements are also plausible. The infant oral microbiome mirrors the trend observed in the stool, with the initial presence of species common to multiple maternal body sites, rapidly followed by the predominance of species more typically associated with oral microbial communities, at 3 days of life (T2; Figure 2B).

### Mothers Transmit a Substantial Fraction of the Strains from Shared Species

**Figure 3:**
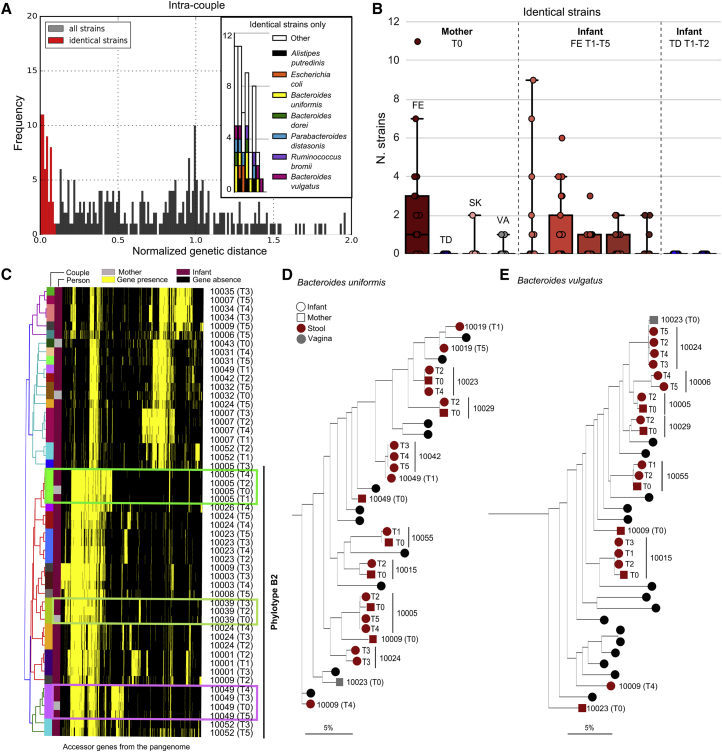
Strain Transmission between Mothers and Their Infants (A and B) The distribution of the normalized strain intra-pair distances (STAR Methods) (A) and the number of vertical transmitted strains for each maternal source and each infant recipient body site and time point (B). (C) *Escherichia coli* strain-specific gene content as identified by PanPhlAn. Strains with clear evidence of vertical transmission are indicated with boxes. (D and E) Mother-infant phylogenies for *Bacteroides uniformis* and *Bacteroides vulgatus* as inferred by StrainPhlAn. Maternal body sites are represented by squares and infant body sites by circles. Mother-infant pairs with at least two samples are labeled with the pair ID in the trees (black circles otherwise).

To add support to vertical mother-to-infant transmission events, it is necessary to identify the same strain variants within the mother-infant pairs. Because considerable individualized strain-level heterogeneity has been observed in the human microbiome (Franzosa et al., 2015, Schloissnig et al., 2013, Truong et al., 2017), finding the same strain in the mother and in the infant would give strong evidence of intra-pair transmission. We thus implemented a novel combination of metagenomic strain-profiling tools (STAR Methods), expanding on methods validated previously (Asnicar et al., 2017). By coupling single-nucleotide variant (SNV) profiling (Truong et al., 2017) with gene-content-based profiling (Scholz et al., 2016), we characterized all the strains with sufficient coverage and applied a conservative threshold (0.1 in the normalized phylogenetic distance; STAR Methods) to call strain identity across paired mother-infant samples and infer vertical transmission (Figure 3A). Although there is no current consensus on the definition of a microbial strain (Segata, 2018), here we adopt the operational definition outlined elsewhere (Truong et al., 2017). This defines strain identity based on genomic identity using a similarity threshold on the SNV rate or gene content, which is consistent with short-term intra-subject strain variation (after excluding strain replacement), while also accounting for sequencing noise and non-adaptive low-frequency variants (Truong et al., 2017).

With this approach, we detected a total of 52 strains shared within mother and infant pairs (out of 317 cases of shared mother-infant species with typable strains), i.e., a 16.4% strain transmission rate. By comparison, we found 46 strains in infants that were in common with unrelated mothers out of a total of 6,319 mother-infant inter-pair shared species. Strain sharing between infants and unrelated mothers is thus a rare event, with a sharing rate of 0.73%. This rate is even lower than we previously observed for strain sharing among an adult worldwide population (Truong et al., 2017), which was based solely on SNV-based profiling, indicating that the combined approach of gene content and SNV strain profiling used here is more conservative. Overall intra-pair strain sharing was >22-fold higher than inter-pair sharing (Fisher test, p < 1 × 10^−15^), demonstrating the influence of the maternal source in shaping the infant microbiome.

### The Maternal Gut Microbiome Is the Source of the Majority of Transmitted Strains

Considering the maternal sources of transmission, the gut microbiome was the largest donor of the infant-acquired strains (Figure 3B). Common strains were also found in the maternal skin and vaginal microbiomes, but to a lesser extent. The least important route of transmission appears to be the oral cavity, with little evidence of strain sharing between mother and infant. The number of strains in common between mother and infant gradually decreases over time, with 23 at T1 (1 day) and 28 at T2 (3 days), compared with 10 at T3 (1 week), 10 at T4 (1 month), and 6 at T5 (4 months). This supports the hypothesis of selection for niche-specific bacteria from the pool of maternal strains seeding the infant gut.

Among the species for which we observed mother-infant transmission, *Escherichia coli* can be tracked very effectively using pangenome analysis (Scholz et al., 2016) because of its genomic plasticity and large set of accessory genes. Transmission and persistent colonization by *E*. *coli* strains were evident in three cases (Figure 3C) all belonging to the *E*. *coli* B2 phylotype, known for enhanced persistence in the infant gut (Nowrouzian et al., 2006). While other *E*. *coli* phylotypes were present in infants, none of these were shared with their mothers (Figure 3C), which is suggestive of a non-maternal source for these strains. Other typically gut-associated species belonging to bifidobacteria (Figure S4) and *Bacteroides* (Figures 3D and 3E) displayed clear maternal routes of transmission confirmed by SNV identity patterns. As a validation for inferring strains from metagenomes, a comparison was made with strains cultivated from sample aliquots for a subset of the mother-infant pairs. Metagenomically inferred strains were near identical (>99% nucleotide identity) to those identified via single isolate sequencing (Figure S5). Cultivation also confirmed that breast milk is yet another maternal source of bacterial strains colonizing the infant, with two genomes of *B. longum* and *Bifidobacterium bifidum* strains isolated from the same milk sample (STAR Methods) recovered in the corresponding infant stool (Figure S5).

### Vertically Transmitted Microbes Are More Likely to Be Stable Colonizers

**Figure 4:**
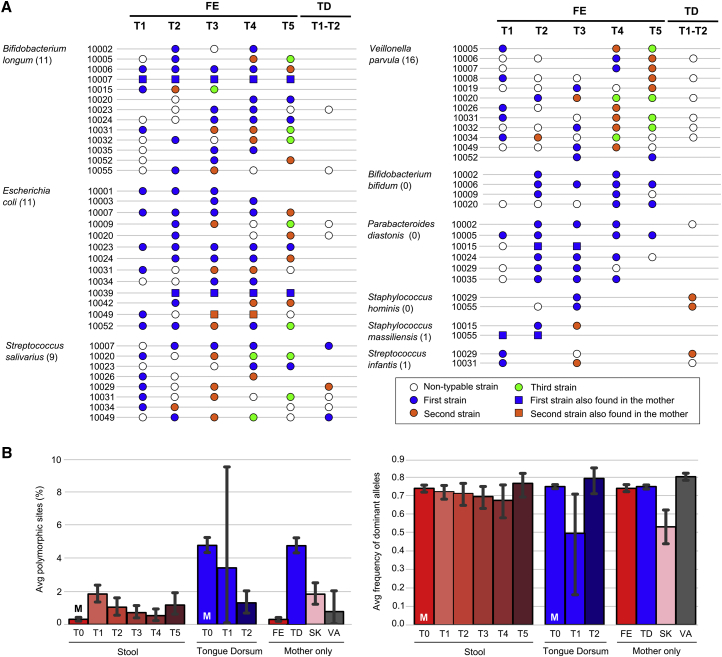
Strain Persistence, Strain Replacement Events, and Strain Heterogeneity (A) Map of the strain dynamics in longitudinal infant stool (FE) samples for selected species (for full map, see Figure S6). The tongue dorsum (TD) column shows the species for which at least one of the strains found in stool was also present on the tongue dorsum. Blue circles represent the first strain of the species identified in the infant, whereas orange and green circles denote the second and third longitudinally identified strain, respectively. Empty circles refer to species for which strain profiling was not possible in the specific sample. Missing samples and samples lacking the species are not reported. The total number of infant replacement events observed in each species is shown in parentheses. (B) Mean percentages of polymorphic sites and average frequency of the dominant alleles in polymorphic sites for each body site and time point (“M” indicates maternal samples). Color coding is as per Figure 1. p values are reported in Table S4. Error bars refer to 95% confidence intervals.

Vertical microbial transmission from the mother to the infant can either be transient or lead to longer-lasting colonization of the infant gut (Korpela et al., 2018). Of the vertically transferred strains, 17 were identified at more than one time point in the infant (Figures 4A and S6). In 12 of these 17 cases, after the first occurrence of the maternally acquired strain we found no subsequent replacement by another conspecific strain, i.e., 70.5% of the strains were retained and 29.5% replaced. In contrast, the 163 strains present in the infant at more than one time point, but without evidence that the mother was the source, were found to be replaced in 119 cases (73% replacement) and retained in 44 (27% retention). Vertically transmitted strains therefore seem to have a better fitness for colonization than strains without evidence of acquisition from the mother (70.5% versus 27% stable colonization, Fisher test, p < 0.001). This supports the intriguing hypothesis that maternal strains are likely to be more ecologically adaptable in the infant compared with non-maternal strains.

### Conspecific Strain Diversity within Fecal Species Is Higher in the Infant Than in the Mother

We next investigated the total strain heterogeneity for each species in the microbiome of the infants compared with that of the mothers. To estimate conspecies strain heterogeneity and dominance, we analyzed the number of polymorphic nucleotide positions in the single-copy marker genes of each detected strain, as well as the average frequency of the dominant allelic variant in polymorphic positions. The analysis of maternal gut samples confirmed that the adult human gut tends to harbor only one strain of a given species (Truong et al., 2017), with an average fraction of polymorphic sites of 0.31% (Figure 4B). The infant gut microbiome at day 1 (T1) instead has a very high conspecific strain heterogeneity with 6.1-fold more polymorphisms than the mother (p = 1 × 10^−7^). As observed, the early infant microbiome at day 1 postpartum is characterized by a high species diversity (Figure 1B), which is thus also accompanied by a high strain diversity, further suggesting that the pioneering microbiome is a complex community of microbes shaped by the process of ecological selection over time. Correspondingly, at later time points there is a decrease in the intra-species polymorphic rates up to 1 month (T4), to levels comparable with those of the mothers (no significant difference at 1 month compared with the mother). Simultaneously, a higher relative frequency of the dominant strain is observed (Figure 4B). Samples collected from the infant at 4 months of age (T5) then suggest that the strain diversity is increasing and remains significantly higher than the diversity in the mothers (p = 0.0014), potentially as a consequence of the increased exposure of the infant to other possible sources of microbial seeding from the environment. Comparing the conspecific strain diversity of the infant over time with the other maternal body sites (Figure 4B), we identified markedly different levels of heterogeneity (Table S4), with the maternal tongue dorsum significantly more strain diverse than the infant gut (p < 1 × 10^−10^ for all time points). The maternal skin and vaginal microbiomes have instead a strain diversity in line with that of the infant stool (Table S4). Interestingly, and in contrast to the stool, the maternal oral strain diversity compared with infants is significantly higher (p = 2 × 10^−9^ at T2, t test). Nevertheless, in the infant oral cavity, we identified the same pattern observed in the gut, namely a high species and conspecific strain diversity (Figures 1B and 4B) followed by a rapid decline in species and strain heterogeneity due to selection, which is observed to start after a few days postpartum.

### Oral Bacteria Seed the Gut Microbiome in Infants to a Greater Extent Than in the Mothers

**Figure 5:**
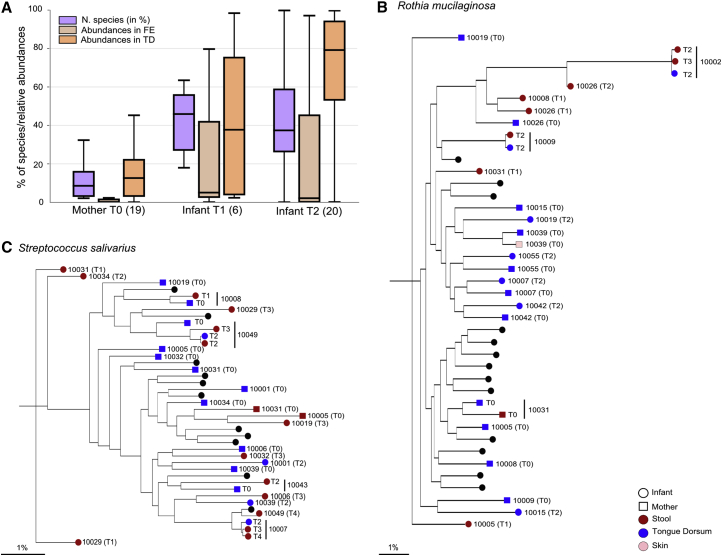
Infants Have More Shared Species and Strains between the Oral and Gut Microbiome Than Their Mothers (A) Number of shared species normalized by the total number of species present in the gut and the tongue dorsum of each subject, and the cumulative abundances of shared species in the two body sites. The number of samples considered for the analysis is reported in parentheses. (B and C) Transmission trees for *Streptococcus salivarius* (B) and *Rothia mucilaginosa* (C). Only pairs with more than two samples present in the tree are shown (black circles otherwise).

The oral cavity is the gateway to the gastrointestinal tract, but the analysis of the oral and gut microbial communities in adults has shown them to be distinct with minimal overlap (Human Microbiome Project Consortium, 2012b, Lloyd-Price et al., 2017). To our knowledge, the extent of species and strain sharing in the oral and gut of infants has not been previously characterized. In our cohort, we found an average of 9.8 species in common between the stool and the tongue dorsum samples in the infant at day 1 (T1) and 7.2 species at 3 days of life (T2). As a baseline of comparison, the average in the mothers was significantly lower with only 5.5 shared species (p < 0.001 for the two time points T1 and T2; Figure 5A). In both infants and mothers, the common species were more abundant on the tongue dorsum compared with the stool (Figure 5A). However, while for the mothers almost all oral-gut shared species were at very low abundance in the stool compared with the tongue dorsum (average of 0.96%), in contrast in the infant they represented about a quarter of the total abundance of the stool microbiome (average of 24.85% and 27.09% for T1 and T2, respectively). This suggests that the oral-gut axis in infants is a rather permissive interface, and that continuous seeding of the infant gut via the oral cavity is more relevant in infants than in adults, perhaps as a consequence of reduced acid production in infants at birth as the gastrointestinal tract develops. Interestingly, the number of shared oral-gut species already decreased in the infant by day 3; however, the abundance of the shared species increased in the oral cavity, suggesting that the orally acquired species at least transiently colonize the gut.

In the infant, the most commonly shared oral-gut species at day 1 (T1) were *Gardnerella vaginalis*, *Propionibacterium acnes*, *Prevotella bivia*, *Atopobium vaginae*, and *Prevotella melaninogenica*, while at day 3 (T2) they were mainly *Rothia mucilaginosa*, *Streptococcus parasanguinis*, and *Streptococcus salivarius* (Tables S5A–S5D). When looking at strain identity within the shared oral-gut species, we found that *S. salivarius* (Figure 5C) and *R. mucilaginosa* (Figure 5B) were the species with the highest number of shared strains (Table S5E). These results suggest that *S. salivarius* and *R. mucilaginosa* might have an increased capacity to survive in both the oral cavity and the gut, at least for a limited time.

### Strains Belonging to as yet Uncharacterized Species Are Also Vertically Transmitted

**Figure 6:**
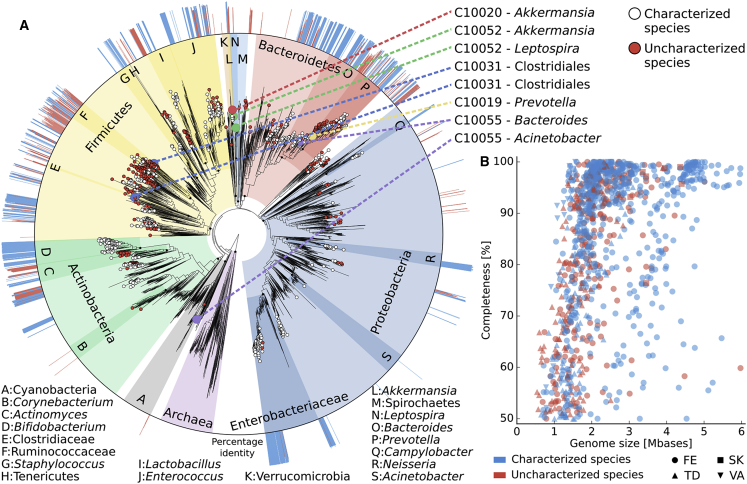
Phylogenetic Placement of 1,132 Metagenomically Reconstructed Genomes and Mother-to-Infant Transmission of Taxonomically Uncharacterized Strains (A) We used PhyloPhlAn2 (Segata et al., 2013) to place the genomes reconstructed with metaSPAdes (Nurk et al., 2017) and binned with MetaBAT2 (Kang et al., 2015) (STAR Methods) on the microbial “Tree of Life” (Ciccarelli et al., 2006, Segata et al., 2013), which encompasses 4,000 species with available reference genomes. Leaf nodes without circles refer to reference genomes from known species, white circles indicate reconstructed genomes that are close (>95% identity) to a known species, and red circles show reconstructed genomes that cannot be assigned (<95% identity) to known species. The eight events of mother-to-infant transmission of strains from species yet to be described are called out on the top right, and the external ring of the phylogeny reports the percent identity of each leaf node against the closest genomes from known species (values below 95% are shown in red). (B) The reconstructed genomes with completeness >50% from each body site are plotted with the corresponding completeness and genome size.

To perform strain profiling for microbes belonging to poorly characterized species without available genomes, we expanded our analysis by performing metagenomic assembly (Nurk et al., 2017) on each sample followed by contig binning (Kang et al., 2015), phylogenetic profiling (Segata et al., 2013), and whole-genome strain identity inference (STAR Methods). Overall, we reconstructed 1,132 metagenome-assembled genomes (on average five per sample; Table S6A) with sufficient quality (≥50% completeness, ≤5% contamination) to be amenable for strain tracking. Of these, 763 genomes could be assigned to a known species by applying a 95% percent identity threshold on the whole sequence (STAR Methods) and were therefore not further processed because these strains were captured by the reference-based methods already considered above. However, the remaining 369 genomes (Figure 6; Table S6A) did not belong to any of the 13,575 microbial species for which at least one reference genome is available (STAR Methods), including 36 that could not even be assigned below the level of family. The genera containing most of the unknown species were *Streptococcus* (32 genomes), *Clostridium* (31), and *Prevotella* (31).

Next, we compared the 369 taxonomically uncharacterized genomes against each other to identify the presence of the same strain in different metagenomic samples. Using a strict threshold of 99.5% identity over the full length of the genomes, we identified eight vertical transmission events (Figure 6; Table S6B). In six cases the strain sharing was between the mother and infant gut microbiomes. Two of these strains belonged to uncharacterized species in the *Akkermansia* and *Bacteroides* genera (less than 89% identity with the closest available genomes over less than 75% of the length), while for the other four strains classification was even more challenging and we could only infer that they belonged to four different phyla (Verrucomicrobia, Proteobacteria, Bacteroidetes, and Firmicutes; Table S6B). In addition to the fecal transmission routes, two vertical transmission events were also observed from other body sites with an uncharacterized Clostridiales strain (99.9% of similarity) shared by the maternal vaginal community and the stool of the infant, and an unknown *Leptospira* strain (99.9% of similarity) shared by the skin microbiome of the mother and the saliva microbiome of the infant. There was only one case of a strain from an unknown species with 99.9% similarity within an unrelated mother-infant pair, strongly confirming the occurrence of vertical transmission for the eight genomes above (Fisher test, p < 1 × 10^−9^) and confirming that uncharacterized species have a role in the mother-to-infant microbial seeding.

## Discussion

We investigated here the early acquisition and development of the infant gut and oral microbiomes, and in particular the role of different maternal sources in this process, by means of a longitudinal multiple body site metagenomic approach. Among our main findings is the very high microbial diversity and strain heterogeneity in the pioneering infant gut microbiome even in the first day of life, which dramatically decreases within the first week before recovering and gradually increasing over the next 4 months. While we cannot discount the possibility of intra-uterine microbial acquisition, this suggests early seeding with an overall species diversity and strain heterogeneity far higher than previously appreciated, followed by steep selection forces that maintain only part of this early biodiversity. The selection process is corroborated by the decreasing strain heterogeneity in the developing infant microbiome and the initial increase of facultative anaerobes, which are subsequently replaced by strict anaerobes consistently with the biochemical changes of the infant gut environment. Far from being a static process, microbial seeding from maternal sources is continuous, with some species and strains appearing in the infant at later time points. We thus describe how the microbial colonization process in the infant reflects a balance between influx of microbial strains and a process of niche selection. This balance is likely the key for the physiological development of the infant microbiome and should be further studied to unravel potential links with pathologies in childhood.

Another key finding of our study is that we identify microbial strains present in the infants for which there is strong evidence of transmission from their mothers, and that these strains are more likely to adapt to and persist in the infant gut than non-maternally acquired strains. This reinforces the importance of this vertical mother-to-infant microbial transmission from multiple sources, because even though the maternal microbiomes cannot explain many microbial strains present in the infant, the transmitted strains appear crucial in the developing microbiome (Korpela et al., 2018). The mechanisms of this phenomenon should be further investigated and may be related to a combination of prebirth transmission, shared environmental factors, and common genetic factors that could partially explain the mother-infant strain specificity. Nevertheless, if we consider vertical microbial transmission as a physiological process under evolutionary pressure in recent human history, the study of vertically transmitted strains can provide the basis for better understanding the impact of non-vaginal birth (C-section) and non-exclusive breastfeeding.

The methodological approaches in this work are a novel combination of reference-based and assembly-based computational profiling that enables us to comprehensively describe mother-to-infant strain transmission and strain-level dynamics in the infant microbiome. The adoption of computational profiling tools with a resolution at the level of individual strains is key because no direct evidence of microbial transmission can be inferred when bacteria are categorized solely at the species level. In this work, we exploit and model the very high inter-subject variability of strains in the general population, and suggest transmission only for the cases in which a mother and her infant have a strain similarity substantially higher than that found between unrelated subjects. Overall, we find 62 cases with strong evidence of strain transmission, eight of which are from species that are currently uncharacterized. We also find evidence of strains originating from multiple maternal sources, with the vaginal, skin, oral, and gut communities all contributing to the early infant microbiome. However, even after a few days postpartum, the contribution of the vaginal and skin microbiome already decreases. Here we focus on the mother, perhaps the most important familiar relationship in the development of the infant microbiome. However, the same techniques can be applied to describe other routes of transmission, including family members other than the mother (Korpela et al., 2018), and the hospital rooms (Brooks et al., 2017). These sets of microbial sources have not been studied together, and their integrated analysis will further contribute to understanding the mechanisms of early microbiome acquisition and subsequent development.

## STAR★Methods

### Key Resources Table

**Table:** 

REAGENT or RESOURCE	SOURCE	IDENTIFIER
**Chemicals**, **Peptides**, **and Recombinant Proteins**		

Man-Rogosa-Sharp (MRS)	Scharlau Chemie, Barcelona, Spain	N/A

**Critical Commercial Assays**		

PowerSoil DNA Isolation Kit	MoBio Laboratories Carlsbad, USA	N/A
NexteraXT DNA Library Preparation Kit	Illumina, California, USA	N/A

**Deposited Data**		

Raw sequencing data	This paper	NCBI-SRA BioProject number PRJNA352475
Assembled contigs	This paper	https://www.dropbox.com/s/7pygii8khtrriw0/genome_bins.tar.bz2?dl=0

**Software and Algorithms**		

FastqMcf	Aronesty, 2011	https://github.com/ExpressionAnalysis/ea-utils/blob/wiki/FastqMcf.md
BowTie2	Langmead and Salzberg, 2012	http://bowtie-bio.sourceforge.net/bowtie2/index.shtml
trim_galore	N/A	http://www.bioinformatics.babraham.ac.uk/projects/trim_galore/
MetaPhlAn2 (version 2.6)	Segata et al., 2012, Truong et al., 2015	https://bitbucket.org/biobakery/metaphlan2
Seqtk	N/A	https://github.com/lh3/seqtk
PanPhlAn	Scholz et al., 2016	https://bitbucket.org/CibioCM/panphlan/wiki/Home
StrainPhlAn	Truong et al., 2017	https://bitbucket.org/biobakery/biobakery/wiki/strainphlan
RAxML	Stamatakis, 2014	https://sco.h-its.org/exelixis/web/software/raxml/
metaSPAdes (version 3.10.1)	Nurk et al., 2017	http://bioinf.spbau.ru/en/spades3.7
MetaBAT2 (version 2.12.1)	Kang et al., 2015	https://bitbucket.org/berkeleylab/metabat/wiki/Home
CheckM (version 1.0.7)	Parks et al., 2015	https://github.com/Ecogenomics/CheckM/wiki
BLASTn (version 2.6.0)	Altschul et al., 1990	https://blast.ncbi.nlm.nih.gov
pyani (version 0.2.6)	N/A	https://github.com/widdowquinn/pyani
PhyloPhlAn2	Segata et al., 2013	https://bitbucket.org/nsegata/phylophlan/wiki/phylophlan2
GraPhlAn	Asnicar et al., 2015	https://bitbucket.org/nsegata/graphlan/wiki/Home
Samtools	Li, 2011, Li et al., 2009	http://samtools.sourceforge.net/

### Contact for Reagent and Resource Sharing

Further information and requests for resources, reagents, and software should be directed to and will be fulfilled by the Lead Contact, Nicola Segata (nicola.segata@unitn.it).

### Experimental Model and Subject Details

#### Experimental Design and Cohort Recruitment

A total of 25 pregnant women (26-43 years of age, BMI before pregnancy between 16.5 and 29.4) were recruited by Santa Chiara Hospital in Trento, Italy, between April 2015 and July 2016. The protocol of this study was approved by the Ethics Committee of Santa Chiara Hospital (Trento, Italy) and the Ethics Committee of the University of Trento. Parents were asked to provide informed consent and complete questionnaires regarding pregnancy data, maternal diet, medical records, and lifestyle before and during the pregnancy (Table S1A).

Exclusion criteria included delivery by Caesarean section, pre-term birth (<37 weeks), body temperature >38°C, birth weight <2.5 Kg, antibiotic treatments during pregnancy, intention to avoid formula feeding at least for the first six months. The infant feeding practices as well as antibiotic usage and complete medical records across the sampling period were also collected (Table S1A). At 4 months of age, 56% of infants were exclusively breastfed (14 out of 25), while 12% (3 out of 25) of mothers reported mixed feeding and 16% exclusive formula feeding (4 out of 25). For four pairs, the sampling was interrupted before the age of 4 months, and therefore no metadata in terms of feeding are available.

### Method Details

#### Sample Collection

The samples collection procedure was based on HMP sampling guidelines (Human Microbiome Project Consortium, 2012b). Skin samples were collected using Catch-All-Swabs (Epicentre Technologies, Wisconsin, USA) shortly after birth but before the skin-to-skin contact with the infant, by swabbing the upper area of the maternal breast (intermammary cleft). After pre-moistening with 2 ml SCF-1 buffer (50 mM Tris buffer, pH 7.6, 1mM EDTA, pH 8.0, and 0.5% Tween-20) (Human Microbiome Project Consortium, 2012b) contained in a 15 ml sterile screw top collection tube (Sarstedt, Nümbrecht, Germany), the swab head was rubbed back and forth for approximately 30 seconds over the area (repeating twice) before the swab was returned to the buffered solution. Vaginal swabs were collected before delivery, as soon as possible upon arrival at the delivery ward to reduce potential blood contamination. The swab was rubbed 5 times, with a circular motion, in the vaginal introitus and then the swab head was placed in a 15 ml sterile screw top collection tube containing 2 ml SCF-1 buffer. Maternal tongue dorsum swabs were collected shortly before or during delivery by rubbing a swab on the central area of the back of the tongue for approximately 5 seconds. The swab head was then placed in a 15 ml collection tube containing 2 ml SCF-1 buffer. The same procedure was used for sampling the infant tongue dorsum, at time of the collection of the first stool sample. Maternal breast milk was self-collected by the mothers starting at one day after delivery, using gloves to avoid skin contamination. Stool samples from the mother were collected during or shortly after the delivery by the hospital staff, using collection tubes specific for faecal material (Sarstedt, Nümbrecht, Germany). All infant stool samples were self-collected by the mother, following a detailed protocol. The samples collected directly at the hospital were frozen at -20°C immediately after the collection and moved to a -80°C facility within a week, where they remained stored until further analysis. Additional aliquots were collected and stored in 20% glycerol and kept at -20°C for the cultivation experiments. After leaving the hospital, normally three days after delivery, the mothers performed the collection of the infants’ stool samples at home and put the samples immediately at -20°C, which were then delivered to the hospital staff within 12 hours.

#### DNA Extraction and Sequencing

DNA was extracted using the PowerSoil DNA Isolation Kit (MoBio Laboratories Carlsbad, USA), as described in the HMP protocol (Human Microbiome Project Consortium, 2012b). For the stool samples, a preliminary heating step (65°C for 10 minutes, 95°C for 10 minutes) was performed before extraction. For samples collected via swabbing (skin, oral, and vaginal) the head of the Catch-All Sample Collection Swab was removed and put in the PowerBead Tube. The 2 ml SCF-1 specimen-containing buffer was centrifuged at 1000g for 5 min and added to the PowerBead Tube for cell lysing performed using the MOBIO Vortex Adapter (MO BIO Catalog No. 13000-V1). DNA was recovered in 10 mM Tris pH 7.4 and quantified using Qubit 2.0 (Thermo Fisher Scientific, Massachusetts, USA) fluorometer as per manufacturer's instructions. Sequencing libraries were prepared using the NexteraXT DNA Library Preparation Kit (Illumina, California, USA), following the manufacturer's guidelines. The sequencing was performed on the HiSeq2500 (Illumina, California, USA).

#### Metagenome Quality Control and Preprocessing

Out of the 225 collected samples subjected to sequencing, 216 provided more than 5.1 x 10ˆ9 total reads (average 23.96 x 10ˆ6 reads per sample) and were therefore used for the downstream analysis. Of these samples, 119 (55%) were stool samples (21 from the mothers and 98 from the infants), 15 (6.9%) were skin swabs from the mother, 63 (29.2%) were oral cavity swabs (24 from the mothers and 39 from the infants), and 19 were derived from vaginal swabs (8.8%).

The generated raw metagenomes were processed with FastqMcf (Aronesty, 2011) by trimming positions with quality <15, removing low-quality reads (mean quality <25), and discarding reads shorter than 90 nt. Human and bacteriophage phiX174 (Illumina spike-ins) DNA were then removed using BowTie2 (Langmead and Salzberg, 2012) to map the reads against the reference genomes. The adapters were also discarded by trim_galore (http://www.bioinformatics.babraham.ac.uk/projects/trim_galore/) with parameters "-q 0 --nextera --stringency 5". All samples providing less than 50,000 reads were excluded from the downstream analysis. The number of reads for each sample after preprocessing is reported in Table S1B.

#### Species- and Strain-Level Profiling

Species-level quantitative taxonomic profiling was performed using MetaPhlAn2 (version 2.6) (Segata et al., 2012, Truong et al., 2015) on the post-processed reads. MetaPhlAn2 estimates the relative abundances of each known microbial species. These relative abundances are proportional to the underlying absolute species concentrations that cannot be quantified from metagenomic data alone. Taxonomic profiles included bacteria, archaea, microbial Eukaryotes, and viruses, and were inferred by MetaPhlAn2 using the ∼1 M unique clade-specific marker genes identified from ∼17,000 reference genomes (∼13,500 bacterial and archaeal, ∼3,500 viral, and ∼110 eukaryotic). Rarefaction analysis was performed using Seqtk (https://github.com/lh3/seqtk) at 5 million reads per sample; 57 samples (28 of which from infants) were excluded because of insufficient sequence depth (less than 5 million reads per sample after sub-sampling).

Strain-level analysis was performed using a combination of gene-content-based profiling using PanPhlAn (Scholz et al., 2016), and single-nucleotide variant profiling using StrainPhlAn (Truong et al., 2017). PanPhlAn was applied on the preprocessed metagenomes using default parameters generating a presence/absence gene-family profile independently for each sample and each species present in the reference database. A species-specific gene-family matrix was obtained by combining profiles from different samples and was further processed with RAxML (Stamatakis, 2014) to generate a species-specific phylogenetic tree. Comparison among different trees was performed by normalizing distances in each tree by its median value. Finally, strain distance for any pair of samples was defined as the normalized phylogenetic distance on the corresponding tree. Similarly, StrainPhlAn was run on the preprocessed reads with default parameters and adding the options "-alignment_program mafft" and "--relaxed_parameters3″ for each reference species and by considering pair-specific markers. This generated a maximum of twenty-five (i.e., the number mother-infant pairs) phylogenetic trees for each species. A single species-specific value for each pair of samples was obtained by averaging their distances along the different trees. Strain distance was defined as for PanPhlAn including normalization of each tree by its median value. The final strain distance for any sample pair was obtained by taking the minimum value between the two strain distance values generated by PanPhlAn and StrainPhlAn. We considered a threshold of 0.1 on the strain distance for defining a pair of strains as the same strain. This threshold was chosen as the value dividing the two peaks in the bimodal distribution of all-versus-all normalized species-specific strains distances representing identical and thus possible transmitted strains (values smaller than 0.1) and clearly distinct strains (values bigger than 0.1). The same approach was adopted for all species under the assumption that this threshold is very stringent and that almost identical genomes represent almost identical strains irrespective of the species they belong to. Results reported in Figures 3A and 3B were obtained by considering the minimum strain distance value for each pair of subjects to avoid skewing of the statistics due to effects of multiple timepoints and multiple body sites effects. Strain-level phylogenies presented in Figures 3D and 3E and in Figures 5B and 5C were obtained from the StrainPhlAn analysis using RAxML (parameters: -m GTRCAT –p 1234) (Stamatakis, 2014). For the four phylogenies in the two figures we also reported the proportion of phylogenetic distance in percentage computed with respect to the total branch length of the respective phylogeny.

Results reported in Figure 1C were computed using the Bray-Curtis dissimilarity and the MDS algorithm, both implemented in the scikit-learn Python package (scikit-learn).

#### Profiling by Metagenomic Assembly

All samples were processed independently for *de novo* metagenomic assembly through metaSPAdes (Nurk et al., 2017) (version 3.10.1 using default parameters), discarding contigs shorter than 1000 nt. Reads were mapped to contigs using Bowtie2 (Langmead and Salzberg, 2012) (version 2.2.9; option “--very-sensitive-local”), and the mapping output was used for contig binning through MetaBAT2 (Kang et al., 2015) (version 2.12.1; option “-m 1500”). CheckM (Parks et al., 2015) (version 1.0.7; lineage specific workflow) was applied to the resulting bins, and only those with sufficient quality (≥50% completeness, ≤5% contamination) were considered for further analysis. We used BLASTn (Altschul et al., 1990) (version 2.6.0; default parameters) to map the contigs of each reconstructed bin against all the microbial reference genomes (including bacteria, archaea, viruses, and microeukaryotes) available in the NCBI repository as of September 2016 for a total of 13,575 unique and named species. Each position of a given contig was assigned to the hit with the highest *bitscore*, and then the median percent identity across the mapping positions was computed. The average percentage identity for a genome was computed by averaging the percent identity values amongst its contigs. For detecting potential mother-to-infant transmissions, we considered only bins with an average percent identity below 95%, as genomes with available close references were already considered by the assembly-free approach described above. The 369 genomes without a close reference were then compared against each other to identify the presence of the same strain in different samples. We computed the average nucleotide identity (ANI) using the pyani tool (v.0.2.6; option “-m ANIb”) and considered a threshold of 99.5% identity over the full length of the genomes to define strain identity. Because of the use of the whole genome instead of species-specific markers and pangenomes, the strain identity thresholds for metagenomically assembled strains and StrainPhlAn/PanPhlAn profiled strains are not directly comparable. Phylogenetic placement of the reconstructed genomes was obtained by running PhyloPhlAn2 (Segata et al., 2013), and visualized through GraPhlAn (Asnicar et al., 2015).

#### Integrated Cultivation and Metagenomic Analysis of Bifidobacteria

*Bifidobacterium* strains were inoculated in Man-Rogosa-Sharp (MRS) (Scharlau Chemie) supplemented with 0.05% (wt/vol) L-cysteine hydrochloride and incubated in an anaerobic atmosphere [2.99% (vol/vol) H2, 17.01% (vol/vol) CO2, and 80% (vol/vol) N2] in a chamber (Concept 400, Ruskin) at 37°C for 16 h. Chromosomal DNA was extracted as described previously (Ventura et al., 2001). Library preparation and Illumina sequencing were performed at the GenProbio srl (Parma, Italy) and previously described (Duranti et al., 2017).

We validated the microbial transmission results for bifidobacteria by searching for the presence of the 12 assembled genomes isolated in the metagenomes. We first mapped the metagenomics reads against the 12 isolates genomes using Bowtie2 (Langmead and Salzberg, 2012) (parameters: --very-sensitive-local –k 100000 –no-unal). Then, we performed a SNV-rate by analyzing the “.sam” output from Bowtie2 through Samtools (Li, 2011, Li et al., 2009) and custom Python scripts. For each isolate genome mapped against a single metagenome, we considered the sites with at least two high-quality reads covering the single position in either forward or reverse orientation, as defined in the “DP4” Variant Call Format field.

### Quantification and Statistical Analysis

The permanova analysis has been performed using the scikit-bio Python package (scikit-bio). When not specified otherwise, t-test was used to calculate p-values. All the other statistical analyses have been performed with open source software mentioned and referenced in the description of the analyses. The number and description of the samples are reported in Table S1B, and the statistical analyses always refer to the whole set of samples in the specific condition of interest.

### Data and Software Availability

All metagenomes were deposited and are available at the NCBI Sequence Read Archive (SRA) under BioProject number PRJNA352475 and SRA accession number SRP100409. Taxonomic profiles along with metadata are available in the curated MetagenomicData package (Pasolli et al., 2017). Assembled contigs are available at https://www.dropbox.com/s/7pygii8khtrriw0/genome_bins.tar.bz2?dl=0. All the software packages used in this study are open source and publicly available and the new software developed for this study is contained in the new releases of PanPhlAn (Scholz et al., 2016) and StrainPhlAn (Truong et al., 2017) available at https://bitbucket.org/CibioCM/panphlan/wiki/Home and https://bitbucket.org/biobakery/biobakery/wiki/strainphlan respectively.

## Supplemental Information

Document S1. Figures S1–S6

Table S1. Summary of the Sample Metadata and Sequencing Results, Related to Figure 1 and STAR Methods (A) Metadata for the samples included in the study. (B) Summary of the sequenced samples and resulting number of reads after preprocessing.

Table S2. List of Species Disappearing after the First Day of Life of the Infant, Related to Figure 1 (A) List of bacterial species present only in the infants stool at one day (T1), with relative abundance >0.5, ranked on the number of occurrences across samples. (B) Prevalence of species that disappeared after T1 (+1 day) with abundance above 1% in three large infant metagenomic studies.

Table S3. List of Species Typically Associated with Stool, Nasal Cavity and Skin, Vagina, and Tongue Dorsum, Related to Figure 2 Species typically associated with (A) the skin, (B) the stool, (C) the tongue dorsum, (D) the vagina, and their prevalence in other body sites.

Table S4. p Values, Related to Figure 4B (A) p values for Figure 4B on mean percentages of polymorphic sites, using t test. (B) p values for Figure 4B on average frequency of dominant alleles in polymorphic sites, using t test.

Table S5. List of Species and Strains Shared between the Tongue Dorsum and the Gut of the Infants, Related to Figure 5 (A–D) List of most relevant species shared between the tongue dorsum and the stool in the infant at 1 day (T1) and 3 days (T2). (E) List of strains shared between tongue dorsum and gut in infants.

Table S6. Results of the Assembly-Based Profiling Pipeline, Related to Figure 6 (A) Summary of the statistics of the reconstructed genomes. (B) Summary of the statistics about uncharacterized species transmitted from the mother to the infant.

Document S2. Article plus Supplemental Information
