# Supplementary material for: Mother-to-Infant Microbial Transmission from Different Body Sites Shapes the Developing Infant Gut Microbiome
Source: Cell Host Microbe. 2018 Jul 11;24(1):133–145.e5. doi: 10.1016/j.chom.2018.06.005 (PMC6716579; doi:10.1016/j.chom.2018.06.005)
Supplement: Document S1. Figures S1–S6 [file mmc1.pdf]

Cell Host & Microbe, Volume *24*

Supplemental Information

Mother-to-Infant Microbial Transmission from Different Body Sites Shapes the Developing Infant Gut Microbiome

Pamela Ferretti, Edoardo Pasolli, Adrian Tett, Francesco Asnicar, Valentina Gorfer, Sabina Fedi, Federica Armanini, Duy Tin Truong, Serena Manara, Moreno Zolfo, Francesco Beghini, Roberto Bertorelli, Veronica De Sanctis, Ilaria Bariletti, Rosarita Canto, Rosanna Clementi, Marina Cologna, Tiziana Crifò, Giuseppina Cusumano, Stefania Gottardi, Claudia Innamorati, Caterina Masè, Daniela Postai, Daniela Savoi, Sabrina Duranti, Gabriele Andrea Lugli, Leonardo Mancabelli, Francesca Turroni, Chiara Ferrario, Christian Milani, Marta Mangifesta, Rosaria Anzalone, Alice Viappiani, Moran Yassour, Hera Vlamakis, Ramnik Xavier, Carmen Maria Collado, Omry Koren, Saverio Tateo, Massimo Soffiati, Anna Pedrotti, Marco Ventura, Curtis Huttenhower, Peer Bork, and Nicola Segata

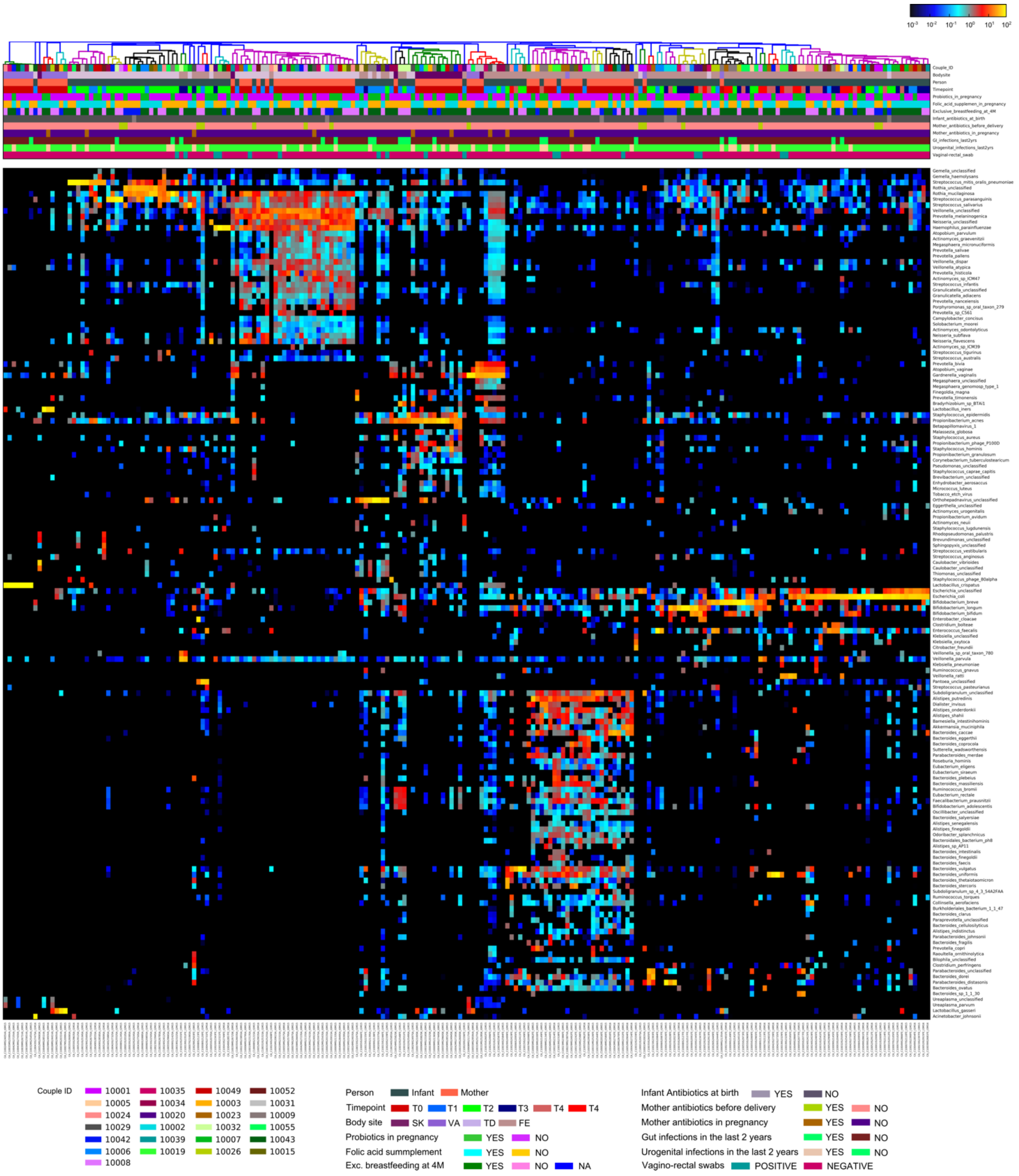

Figure S1. Related to Figure 1. Taxonomic composition and abundance at the species level of all collected samples. Collected metadata include probiotics, folic acid and antibiotics consumption during pregnancy, antibiotics administered to the mother immediately before the delivery, antibiotics administered to the infant shortly after birth, gut and urogenital infections in the mother in the past 2 years, result of the vaginal-rectal swab (standard hospital practice aimed at identifying the presence of *Streptococcus* species belonging to the group B), and breastfeeding practice at 4 months.

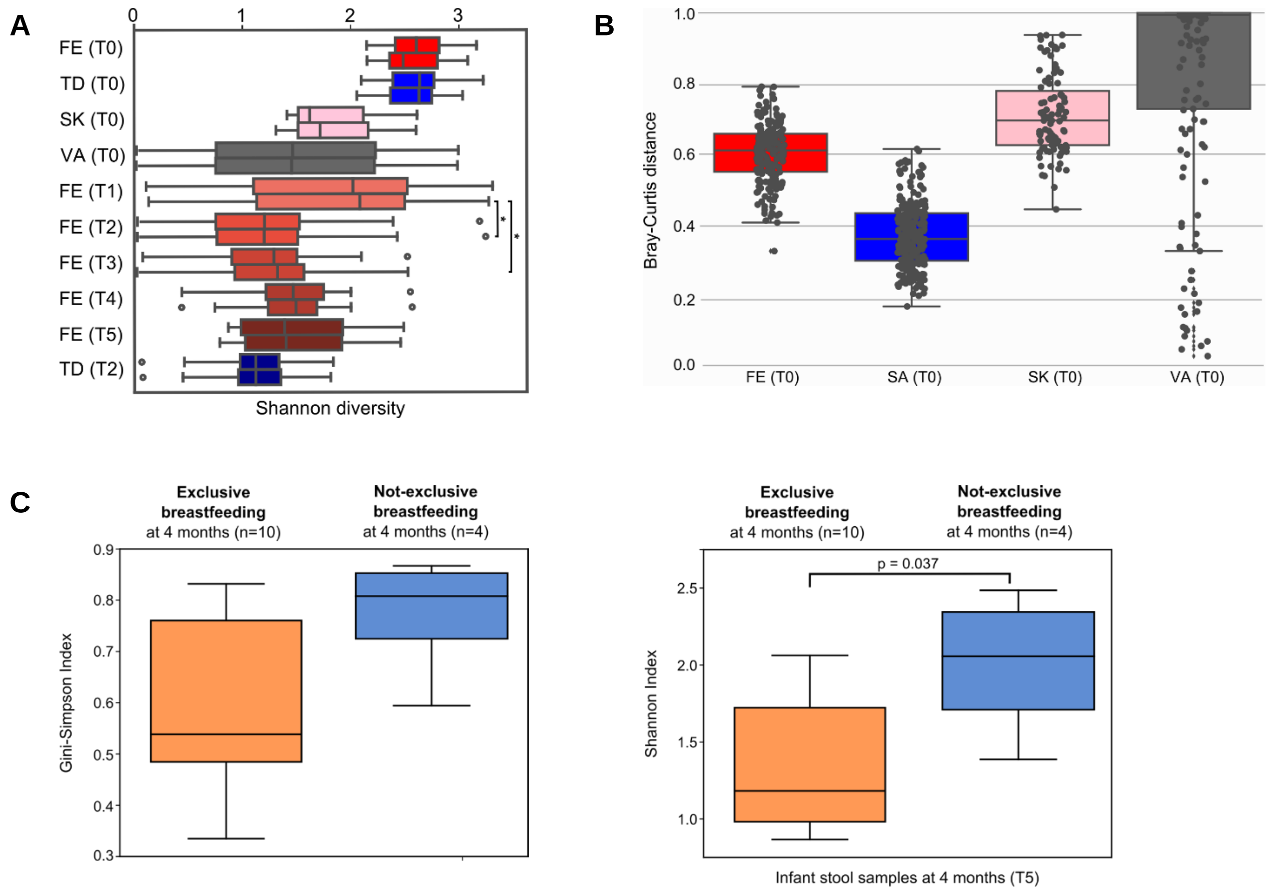

Figure S2. Related to Figure 1. Diversity measures of the sequenced metagenomes. (A) Comparison between standard (upper boxplot) and rarefied (lower boxplot) Shannon alpha diversity for each timepoint and body site. Out of 216 samples, 159 were above the rarefied threshold of 5 million bases. All the tongue dorsum samples at day one (T1) and 5 at day three (T2), 5 stool samples at day one (T1), 1 at one week (T3), 1 at 4 months (T5) and some vaginal, skin and tongue dorsum swabs from the mother (16, 12 and 1 respectively) were discarded in the process. We calculated the Shannon alpha diversity on the original data without rarefaction (upper boxplot) and the rarefied Shannon alpha diversity (lower boxplot) for each timepoint and body site. The * indicates a $p < 0.05$ (t-test). (**B**). **Inter-mother beta-diversity (Bray-Curtis on log-scaled relative abundances) for each maternal body site (stool, tongue dorsum, skin and vagina) at delivery.** The vaginal microbiome shows a higher inter-subject variability compared to the other maternal body sites, due to the presence of the different vaginal community types in the sampled population. **(C). Gini-Simpson and Shannon alpha diversity for faecal samples from exclusively breastfed and (partially or completely) formula-fed infants at 4 months.** Exclusive formula feeding refers to infants that switched from exclusive breastfeeding to a formula-only based diet between the first and the fourth month of age. Numbers of infants for each category are written in brackets (for 11 couples we did not have faecal samples at T5). Two-sided t-test was used to calculate p-values. Feeding mode data for every infant available in the **Table S1A.**

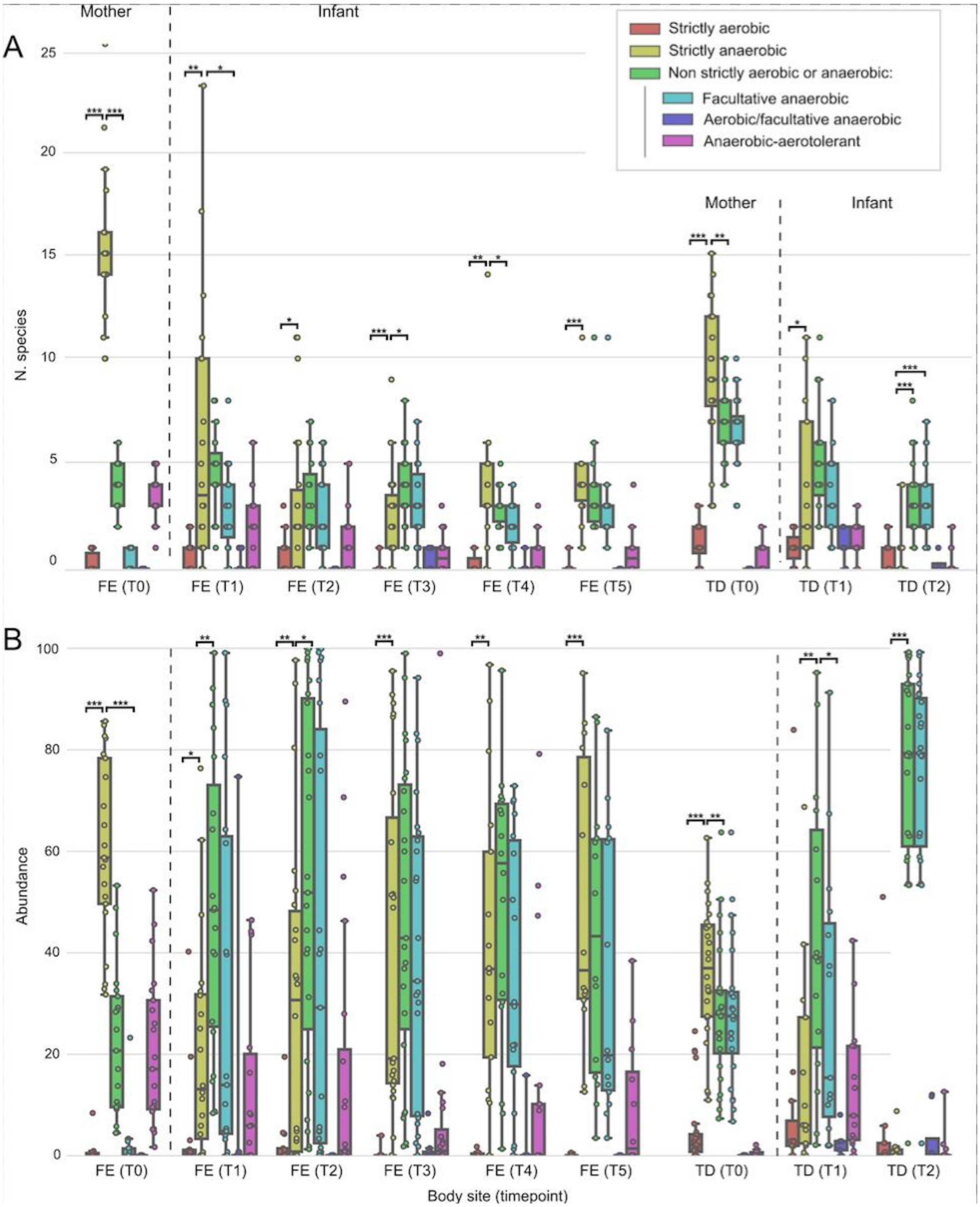

Figure S3. Related to Figure 1 and Figure 2. (A) Number of species and (B) their cumulative abundances present in the stool and oral samples, divided for their ability to utilize oxygen. Species not defined as "Unclassified" by MetaPhlAn2 and present with at least 0.5% of relative abundance were considered. The increase in strict anaerobes abundances, starting from T2 in the faecal samples, is mainly associated to *Bifidobacterium bifidum*, *Bifidobacterium breve*, *Bifidobacterium longum*, *Parabacteroides distasonis,* and *Veillonella parvula*. P-values referred to the aerotolerance classes within the same body site and timepoint are shown in the plot (* *for P-values <0.05, ** for P-values <0.01 and *** for P-values <0.001, t-test)*.

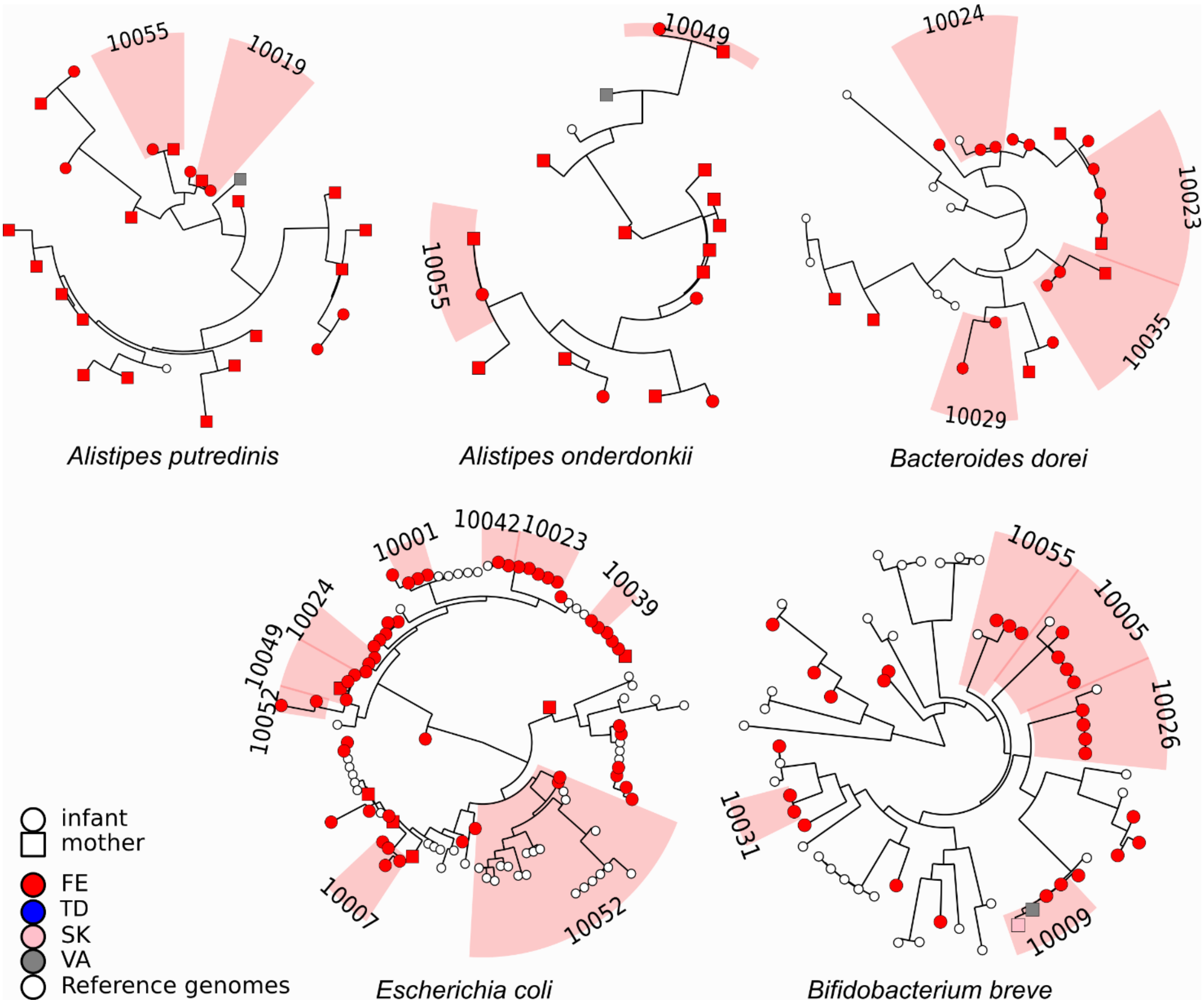

Figure S4. Related to Figure 3. Phylogenetic trees of vertically transmitted species in stool samples. Transmission between paired maternal- and infant- stool samples (represented by squares and circles, respectively) are highlighted with red shaded areas (pair ID is also shown).

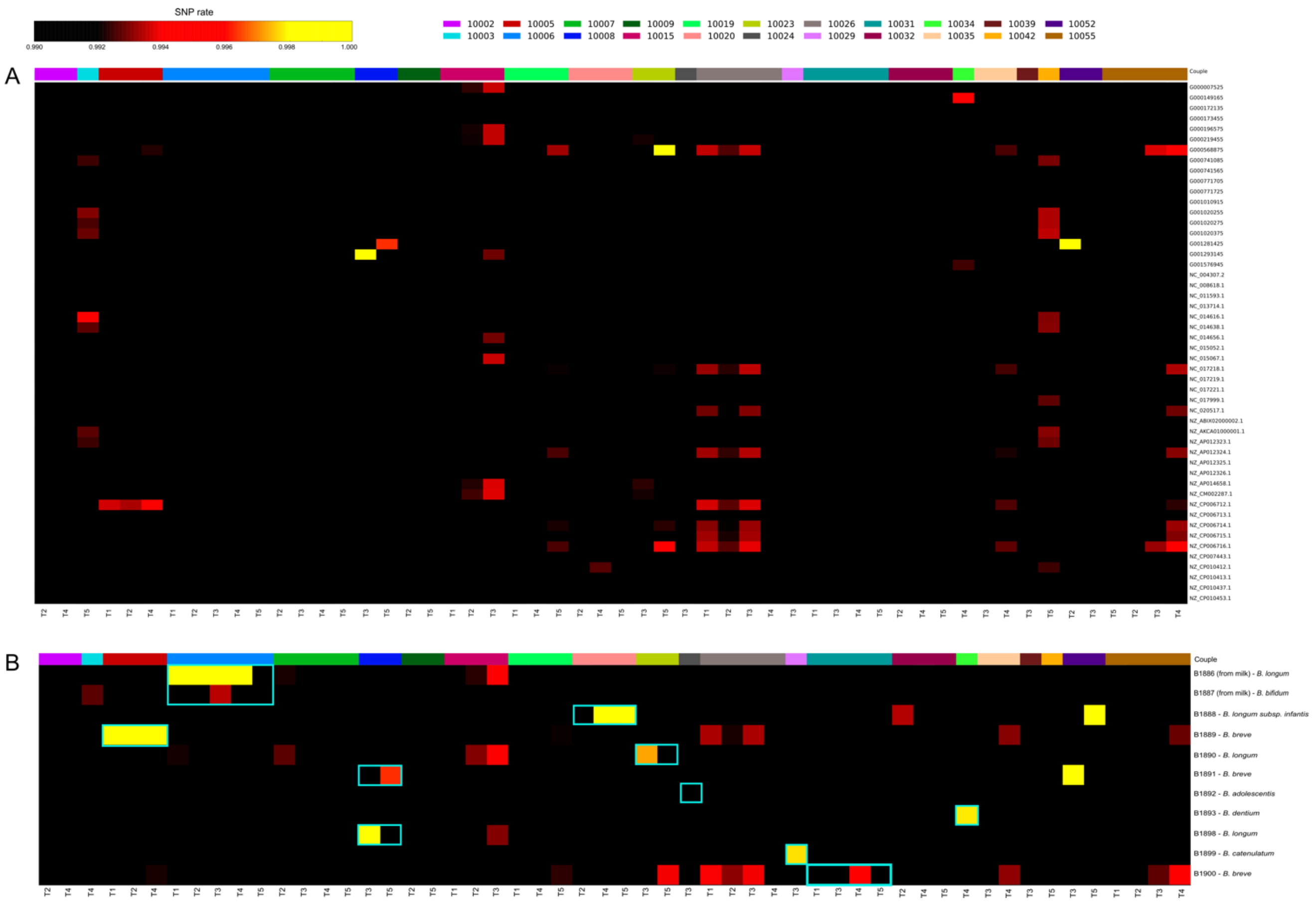

Figure S5. Related to Figure 3. Comparison of metagenomically inferred *Bifidobacterium* strains and Bifidobacteria cultured for a subset of the mother-infant pairs. Panel (**A**) shows the genetic distance between publically available reference genomes and the infant metagenomes. Panel (**B**) shows the genetic distance of the *Bifidobacterium* strains isolated from some of the milk and stool samples of this cohort identified in all the infant stool metagenomes. Cyan squares highlight the infant-mother pairs where the strain was isolated from. Two strains, *B. longum* (B1886) and *B. bifidum* (B1887), were isolated from a single milk sample (pair 10006). The *B. longum* (B1886) isolate was identified in the corresponding infant at four timepoints (>99.99% identity) and the *B. bifidum* (B1887) at one timepoint (>99.5% identity), validating the strain identification directly from metagenomes and the breast milk as a source of infant acquired microbes. Further, nine *Bifidobacterium* strains (belonging to *B. longum*, *B. breve*, *B. adolescentis*, *B. bifidum*, *B. catenulatum,* and *B. dentium* species) were isolated from faecal samples of eight infants. The isolates were identified in infants from eight different pairs, at >99.0% identity, validating again the possibility of identifying strains directly from metagenomes.

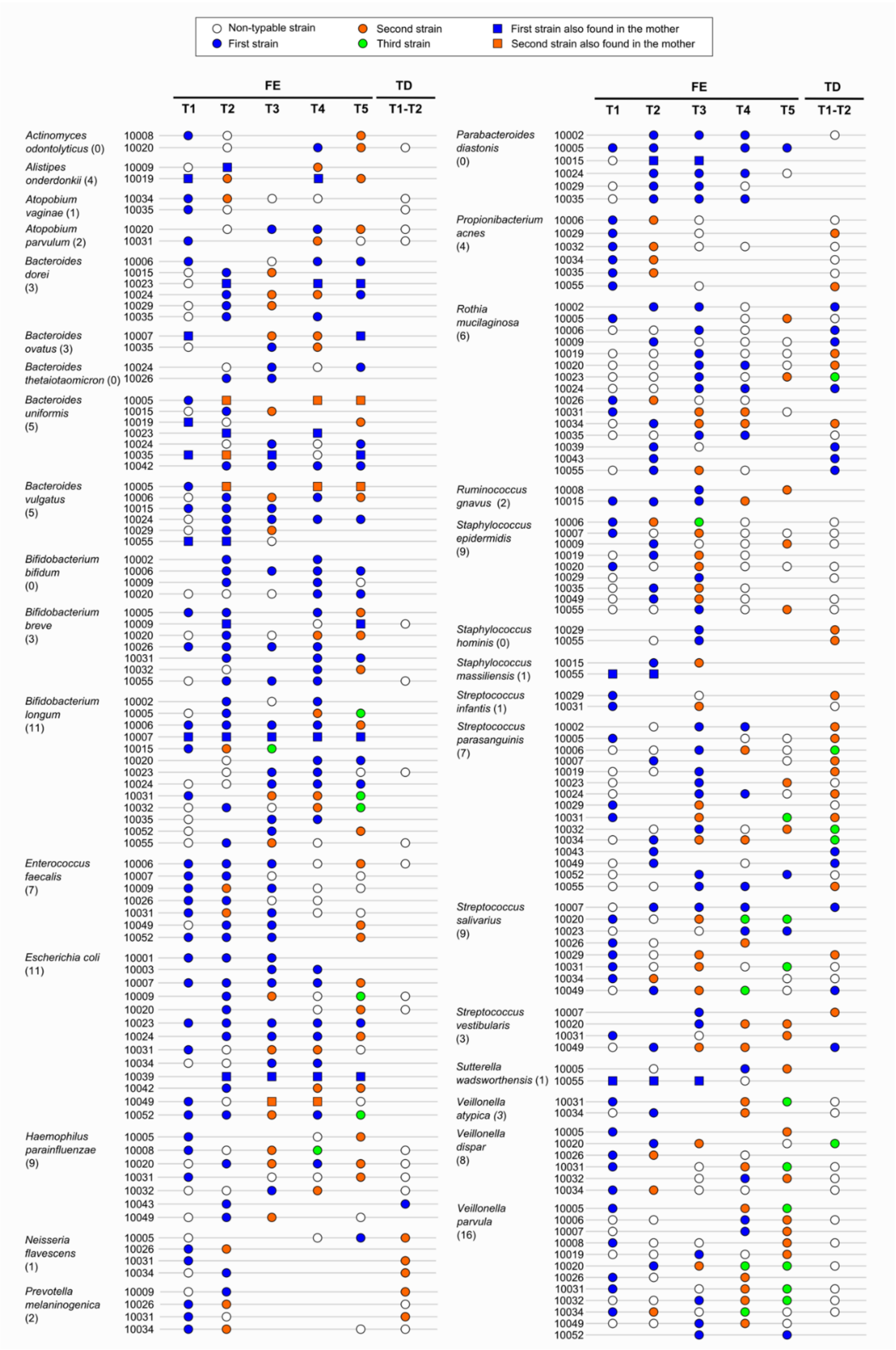

Figure S6. Related to Figure 4. Full list of strain replacement events in the infant body sites. Species with at least two pairs are shown. Empty circle present when only the identification at the species-level was available, i.e. the strain reconstruction was not possible (non-typable strain). Missing circle when the species is not present in the sample. In brackets, the number of replacement events per species (only infants stool samples are considered). In total, we identified 136 replacement events (on average 4.7 events per species and 5.4 per pair).
